# Potassium in Root Growth and Development

**DOI:** 10.3390/plants8100435

**Published:** 2019-10-22

**Authors:** Marek Sustr, Ales Soukup, Edita Tylova

**Affiliations:** Department of Experimental Plant Biology, Faculty of Science, Charles University, Vinicna 5, 128 44 Prague 2, Czech Republic; sustrm@natur.cuni.cz (M.S.); asoukup@natur.cuni.cz (A.S.)

**Keywords:** potassium, root growth, root system architecture, deficiency, KT/HAK/KUP transporters

## Abstract

Potassium is an essential macronutrient that has been partly overshadowed in root science by nitrogen and phosphorus. The current boom in potassium-related studies coincides with an emerging awareness of its importance in plant growth, metabolic functions, stress tolerance, and efficient agriculture. In this review, we summarized recent progress in understanding the role of K^+^ in root growth, development of root system architecture, cellular functions, and specific plant responses to K^+^ shortage. K^+^ transport is crucial for its physiological role. A wide range of K^+^ transport proteins has developed during evolution and acquired specific functions in plants. There is evidence linking K^+^ transport with cell expansion, membrane trafficking, auxin homeostasis, cell signaling, and phloem transport. This places K^+^ among important general regulatory factors of root growth. K^+^ is a rather mobile element in soil, so the absence of systemic and localized root growth response has been accepted. However, recent research confirms both systemic and localized growth response in *Arabidopsis thaliana* and highlights K^+^ uptake as a crucial mechanism for plant stress response. K^+^-related regulatory mechanisms, K^+^ transporters, K^+^ acquisition efficiency, and phenotyping for selection of K^+^ efficient plants/cultivars are highlighted in this review.

## 1. Introduction

Potassium is a macronutrient that may constitute up to 10% of plant dry weight [1]. It is a major inorganic cation in the plant cytoplasm, essential for activity of various enzymes, including those participating in primary metabolism [2]. It contributes significantly to turgor regulation, which is important for many plant processes, such as stomatal function [3,4], cell volume growth [5,6,7], existence of cytoplasm-plasma membrane-cell wall continuum [8], and plant movements [9]. To fulfil the diverse developmental and physiological functions of K^+^ in plants, broad spectrum of K^+^ transporters and channels evolved to orchestrate K^+^ transport [10,11,12,13]. 

Root system growth and development relies on K^+^ at various levels. Protein synthesis and enzyme activity of root cells need adequate cytoplasmic K^+^ levels to maintain the cytoplasmic pH [14] and the anionic charge of proteins [15]. Cell expansion in the elongation zone requires turgor pressure, which builds up via osmotically active substances, including K^+^ [16,17]. In the root maturation zone, root hairs grow apically via the action of K^+^ fluxes [18,19,20]. K^+^ affects R:S ratio (root to shoot biomass partitioning) via phloem transport [21,22]. Moreover, adaptive changes of root system architecture (RSA) and root hair coverage evolved in plants to enhance K^+^ uptake in potassium limiting conditions [10,23]. 

Soil K^+^ bioavailability is often low (especially in acidic soils) and limited mostly to the topsoil as most of soil potassium is incorporated in minerals [24,25]. K^+^ limitation is thus a common problem affecting agricultural production [25,26]. Plants engage high-affinity K^+^ transporters, modulate K^+^ channel transport properties [27,28,29,30], and change root system architecture (RSA) to cope with K^+^ deficiency [31]. Some growth responses to low K^+^ provide functional adjustments of the root system to enhance K^+^ acquisition efficiency. Sensing local K^+^ availability in rhizosphere triggers local root growth [32], but preferential branching to K^+^ rich patches seems to be mild compared to N or P local response [33,34]. K^+^ limitation negatively impacts root elongation and the number of first order lateral roots [35,36,37], but the response varies among species, cultivars, ecotypes, and even root types [35,38]. Suppressed cell volume growth or limited phloem delivery of assimilates to belowground organs may participate in root growth inhibition [21,22]. K^+^ scarcity also increases plant susceptibility to biotic and abiotic stresses [26,39]. 

In this review, we focused on K^+^ involvement in root growth and root system architecture establishment at various levels, from cell growth up to root system response to stress factors. The root system responses to low K^+^ stress are highlighted.

## 2. Potassium and Root Cell Expansion

The crucial role of osmotically active K^+^ cation in turgor-driven cell expansion is well known (see e.g., [5,17,40]). Two voltage-dependent, inward-rectifying *Shaker* K^+^ channels, KAT1 and KAT2 (K^+^ CHANNEL IN ARABIDOPSIS THALIANA1, 2), and K^+^ transporter KT2/KUP2/SHY3 (K^+^ TRANSPORT2/K^+^ UPTAKE2/SHORT HYPOCOTYL3) were shown to participate in auxin-induced cell expansion in the hypocotyls and leaves of *Arabidopsis thaliana* [6,7,41]. *Kup2/6/8* triple mutants display significantly larger leaf epidermal cells, which indicates the involvement of these KT/HAK/KUP (K^+^ TRANSPORT/HIGH-AFFINITY K^+^/K^+^ UPTAKE) transporters in K^+^ efflux and modulation of volume growth [6]. *KT2/KUP2/SHY3* expression is not limited to the shoot. It is expressed in the root tip [7] and might, therefore, be involved in root cell expansion the same way as in the shoot. 

KAT1 functions mostly in the shoot, including in the guard cells [42]; expression of *KAT1* in roots is weak [41]. However, similar *Shaker* K^+^ channel KC1 (K^+^ RECTIFYING CHANNEL1) is active in roots. *KC1* is predominantly expressed in root hairs and the endodermis, and modulates root hair K^+^ uptake [43]. KC1 is a silent regulatory subunit of the heterotetrameric channel AKT1 (ARABIDOPSIS K^+^ TRANSPORTER1), an inward-rectifying K^+^ channel of the *Shaker* family. AKT1 plays a dominant role in root K^+^ uptake over a broad range of K^+^ external concentrations [44,45,46]. The assembly of the AKT1/KC1 heterotetramer is triggered by low external K^+^ availability and modifies AKT1 transport properties so that it prevents K^+^ leakage under these circumstances [29,47]. AKT1 is involved in root hair elongation, but the mechanism is not clear yet [19].

Moreover, a direct link exists between secretion and K^+^ channel activity. SNARE (SOLUBLE NSF ATTACHMENT PROTEIN RECEPTOR; NSF-N-ETHYLMALEIMIDE-SENSITIVE FACTOR) protein SYP121 (SYNTAXIN OF PLANT121), a component of exocytosis regulation machinery controlling vesicle fusion with the target membrane [48], regulates trafficking of K^+^ channel KAT1 and its distribution within plasma membrane subdomains [49]. Moreover, SYP121 directly interacts with the voltage-sensing domain of KAT1 and KC1 channels via FxRF motif and shifts their voltage-driven gating [50,51,52,53]. The SYP121 binding affects the stability of the KAT1 channel in open or closed states and promotes its activity [54]. Binding of SYP121 to KC1 enhances secretion in feedback, highlighting the interplay between exocytosis and K^+^ transport [52,53]. In contrast, binding of the R-SNARE protein VAMP721 (VESICLE-ASSOCIATED MEMBRANE PROTEIN721) to KC1 or KAT1 suppresses their activities [55].

SNARE-dependent control of K^+^ uptake thus presents not only a regulatory pathway of K^+^ acquisition in plants subjected to K^+^ deficiency [23], but also a mechanism to coordinate membrane expansion and cell wall material exocytosis with K^+^ uptake and generation of turgor pressure to drive cell growth [53,56]. SYP121 also coordinates the trafficking of some plasma membrane localized aquaporins (PIP2-7) and thus fine-tunes the water permeability of the membrane [57].

## 3. Potassium and Root Hair Growth

Root hairs are short-lived tubular emergences of rhizodermal cells that serve many important roles in root-soil contact, nutrient uptake, and microbial interactions [58]. Long root hairs are advantageous for K^+^ acquisition under K^+^ shortage, as was shown by comparing different crop species [59] and even rice (*Oryza sativa* L.) genotypes differing in average length of root hairs [60]. 

Root hair establishment starts with the determination of trichoblast cell fate orchestrated by the action of transcription and other regulatory factors. It continues with the formation of a bulge in the cell wall and specialized tip growth of the emerging hair. Many aspects of this process, including trichoblast genetic determinants, cell wall loosening, deposition of new cell wall material, Ca^2+^ signaling, cytoskeleton action, pH gradients, and other processes, have been studied and reviewed thoroughly [58,61,62]. The bulge formation and tip growth require turgor pressure generation, and, here, K^+^ comes to the scene as the main osmotically active inorganic ion of the plant cell [62]. Apoplast acidification was shown to activate K^+^ channels [63] and an acidic environment is typical for bulge outgrowth domains [64]. 

Among others, the AKT1 channel is present in root hairs and participates in K^+^ uptake [44,45,46]. Surprisingly, *akt1* plants exhibit a K^+^-dependent root hair phenotype that is not fully consistent with the proposed role of the AKT1/KC1 channel in triggering root hair growth via K^+^ uptake. *Akt1* mutants possess longer root hairs under zero external K^+^ levels but shorter root hairs under very high K^+^ levels. At 100 mM external K^+^ root hairs of *akt1* mutants completely fail to even elongate. The authors hypothesized that AKT1 negatively regulates root hair elongation in the absence of K^+^, but is required to sustain root hair tip growth under K^+^ levels above 50 mM [19]. 

K^+^ transporter mutant *trh1* (*tiny root hair1*) also shows a strong root hair phenotype. Trichoblast cell fate determination pathways are not affected in *trh1* plants, but root hairs fail to elongate and remain very short [18,65,66]. TRH1/KUP4 is a member of the KT/HAK/KUP family of K^+^ transporters [30,67,68]. Based on a complementation study in *Saccharomyces cerevisiae*, TRH1 can mediate high-affinity K^+^ uptake [18], but the *trh1* root hair phenotype is insensitive to K^+^ supply [18,19]. It can be rescued by external auxin application [69] or phosphorus deficiency, but not iron deficiency [70]. This observation, together with agravitropic root growth, mis-localization of the PIN1 (PIN-FORMED1) auxin efflux carrier, and auxin over-accumulation in the root tip of *trh1* plants, highlights TRH1 function in auxin homeostasis maintenance within root apex as necessary to sustain the growth of root hairs [18,65]. The importance of auxin signaling in root hair growth regulation is evident [71] and supported by root hairs defective phenotypes of other auxin mutants, e.g., *tir1* (TRANSPORT INHIBITOR RESPONSE1), an auxin receptor mutant [72]. However, the effect of TRH1 might be also indirect via modification of K^+^ homeostasis in meristematic cells, as discussed below. 

TRH1 may act as an auxin efflux carrier involved in shoot to root auxin translocation [69]. In roots, it localizes at plasmalemma in a polarized manner. It co-localizes with PIN1 on the basal side of cells in stele in the elongation zone. In the meristematic zone, the preferential localization is in the rhizodermis and cortex. TRH1 might therefore contribute to both acropetal and basipetal auxin transport within the root apex [65] and coordinate environmental cues, auxin signaling, and root hair development [66]. TRH1 seems to function as a homodimer: its assembly is driven by strong interactions between the C-terminal cytoplasmic domains of each of the TRH1 subunits [66]. 

**Figure 1:**
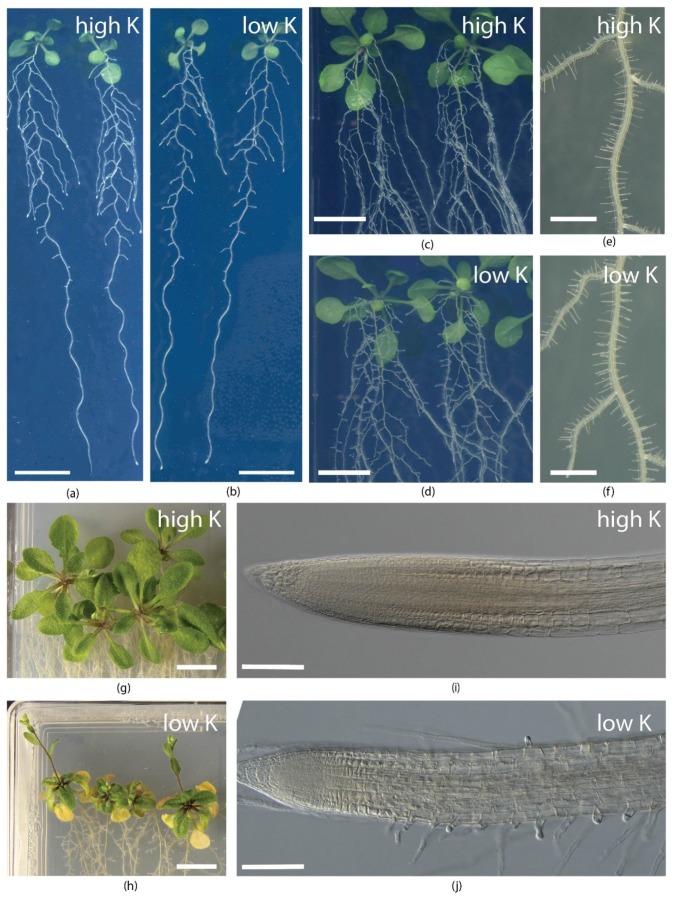
Symptoms of K^+^ deprivation in *Arabidopsis thaliana*: Root systems of 10-day-old in vitro plants on (**a**) high-K and (**b**) low-K media show preferential inhibition of the first-order lateral root growth in low-K. Root system branching of 16-day-old in vitro plants on (**c**) high-K and (**d**) low-K media with enhanced branching to higher orders in low-K. Root hairs of in vitro plants on (**e**) high-K and (**f**) low-K media. Shoots of 16-day-old in vitro plants on (**g**) high-K and (**h**) low-K media with symptoms of K^+^ deficiency on leaves in low-K. Lateral root apex of (**i**) high-K and (**j**) low-K plants. High-K medium: 0.2x strength MS (Murashige and Skoog) with 4 mM K^+^; low-K medium: 0.2x strength MS with 15 μM K^+^. Media were supplemented with 1% agar and 1% sucrose. Scale bars: 1 cm (**a**–**d**, **g**–**h**); 2 mm (**e**–**f**); 100 μm (**i**–**j**).

It must be mentioned that root hair growth responds to K^+^ soil status [73], see Figure 1. K^+^ deficiency stimulates root hair growth [59] in an ethylene-dependent manner [36,74]. This response relies significantly, but not completely, on EIN2 (ETHYLENE INSENSITIVE2), a positive regulator of the ethylene pathway [36,75,76]. K^+^ deficient plants increase ethylene levels [77] and ROS (reactive oxygen species) signaling is involved downstream of ethylene [74,78]. 

## 4. Root System Growth and Architecture under Potassium Deprivation

A common plant response to nutrient deficiency is preferential biomass allocation into the root system (increase in root:shoot ratio) to forage for nutrients from a larger volume of soil [2]. The opposite is often true for low K^+^ stress [79,80]. Although there are also studies showing only a minor effect [59,81], root growth retardation is a common response to K^+^ limiting [79,80]. One possible explanation is the fact that carbohydrates are retained in the shoots of K^+^ deprived plants [21,22,82]. 

Mechanisms to successfully cope with K^+^ deficiency are diverse among species and genotypes. Among the most important are enhanced K^+^ uptake, ability to keep a certain level of root meristem proliferation under low K^+^, chemical modifications of the rhizosphere, and effective K^+^ translocation within the plant body [83]. K^+^-efficient genotypes might combine these strategies [83,84]. For example, some tomato varieties resistant to K^+^ deficiency enhanced uptake capacity per root area, while others enhanced root system growth in comparison to sensitive varieties [85,86]. In soybean, almost no change in RSA was reported, but K^+^ uptake and translocation was considerably higher in K^+^ efficient cultivars [87,88]. Most plants combined both strategies. More intensive root system proliferation was found in K^+^-efficient genotypes in various plant species, including important crops, such as rice [89], maize [81], tobacco [90], and *Medicago truncatula* [91]. Same species are also capable of facilitating K^+^ uptake under K^+^ limiting conditions by activation of high-affinity K^+^ transporters [92,93,94,95,96]. From these data, it becomes clear that enhanced root system proliferation must be supported by more effective K^+^ uptake to some extent.

Change of RSA is not a simple increase of root biomass, but rather a stimulation of branching to higher orders (Figure 1). In a resistant rice genotype, a higher abundance of fine roots within the root system was observed under K^+^ deprivation [80,89]. In *Arabidopsis thaliana* and tobacco, length and number of first order lateral roots decreases, while number of second order laterals increases in K^+^ depleted plants [31,90], see Figure 1. The elongation of the main root seems to be inhibited or unaffected, at least in studies with *Arabidopsis thaliana* (Col-0) as the model plant [35,36,37,77]. The inhibition is well documented, particularly in seedlings [32], while later developmental stages are less sensitive. Thanks to extensive analysis of *Arabidopsis thaliana* ecotypes, large variability in RSA under K^+^ stress has been revealed. The ecotypes can be, with some simplification, classified into two clusters. Ecotypes from cluster I compromise lateral root growth in favor of maintaining growth of the main root (e.g., ecotype Col-0). Ecotypes from cluster II do the opposite, which results in lateral roots overgrowing the tip of the main root [35]. Preferential growth of laterals, branching to higher orders, and long dense root hairs in low-K plants resemble low-P responses and seem reasonable for efficient K^+^ capture from soil, as both Pi and K^+^ are predominantly bioavailable in the topsoil [25,97].

Moreover, localized proliferation of roots to nutrient-rich patches of heterogenous soil is a strategy that enhances nutrient acquisition efficiency. This proliferation is well documented for nitrogen, in both NH_4_^+^ and NO_3_^-^ forms [98,99,100]. For K^+^, which is rather mobile within the soil [24], such a localized response was considered to be absent. This conclusion was in accordance with a study on barley, which did not distinctly respond to localized K^+^ supply, although localized N and P supply triggered massive lateral root growth into the enriched area [33]. Similar results were obtained with soybean in split-root culture [34]. On the other hand, lateral roots of *Arabidopsis thaliana* showed arrested growth on low-K^+^ media when the root system was split between low- and high-K^+^ media [32]. Therefore, localized growth response to K^+^ exists, at least in some species, but appears weak.

Local proliferation of lateral roots into nutrient-rich patches depends on the root’s ability to perceive availability of a particular ion. High-affinity transporters AMT1.3 (AMMONIUM TRANSPORTER1.3) and NRT1.1 (NITRATE TRANSPORTER1.1, also known as CHL1 and NPF6.3) perform this sensoric role for NH_4_^+^ and NO_3_^−^, respectively [98,100]. Analogously, AKT1 and HAK5 (HIGH-AFFINITY K^+^ 5) arise as candidates for K^+^ sensors as these two transport proteins share the majority of K^+^ uptake under K^+^ deficient conditions [27,28,46]. A study by Li and colleagues assigned the sensoric role to AKT1 [32]. In the *Arabidopsis thaliana akt1* mutant, the localized response to K^+^ in media is abolished [32], as well as the proliferation of second order lateral roots in low K^+^ conditions [31]. 

Besides the aforementioned RSA responses, root anatomy might also be affected by K^+^ status of the plant. K^+^-deprived roots may develop more extensive cortical aerenchyma, as was described in maize [101]. This trend seems an adaptive strategy of building “cheap” root biomass to explore more soil. Enhanced cortical senescence also occurs under N and P deficiency and improves growth under nutrient deficiency [97,101,102]. Root endodermal suberization also changes in response to nutrient availability. In *Arabidopsis thaliana*, K^+^ shortage suppresses the suberization and thus affects root transport properties [103,104]. 

## 5. How Does K^+^ Deprivation Regulate Root System Growth and Architecture?

Regulatory mechanisms underlying root growth responses to low K^+^ stress are gradually being unraveled. The inhibition of root growth correlates with limited K^+^ acquisition from environmental and internal K^+^ status. Plants with disrupted high-affinity K^+^ uptake, e.g., *athak5 Arabidopsis thaliana* or *oshak1* rice, have lower tissue K^+^ levels and shorter roots compared to wild-type genotypes in low K^+^ conditions [27,105,106]. Reduced root growth of K^+^-deprived plants was further linked to decreased auxin concentrations in plant tissue [107] and disruption of auxin maxima in the root tip, as observed in *Arabidopsis thaliana* and tobacco [32,108]. In *Arabidopsis thaliana*, degradation of the AtPIN1 transporter was detected [32] and a drop in *NtPIN1* transcription was reported in tobacco [108]. Auxin signaling thus seems to be affected, directly or indirectly, by K^+^ level in the cells of root apical meristem (RAM). Activity of RAM gradually diminishes under low K^+^ stress [35], see Figure 1. Stimulation of RAM activity (e.g., by overexpression of positive regulators [109]) enhances root growth of K^+^ deprived plants. In rice, overexpression of WUSCHEL-related homeobox gene *OsWOX11* stimulated root growth and K^+^ uptake due to larger root surface area, and increased low K^+^ stress tolerance [110]. 

Auxin response machinery is affected by low K^+^ stress as well [107]. Among others, the MYB77 transcription factor is downregulated under K^+^ deprivation [77]. *MYB77* is expressed in primary and lateral roots and MYB77 protein interacts with ARFs (AUXIN RESPONSE FACTORs) to enhance expression of auxin-responsive genes [107]. Lower activity of this auxin response modulator appears to be linked with a decreased number of lateral roots upon K^+^ deprivation. *Myb77* knock-out plants have lower lateral root numbers compared to the wild-type in low K^+^ conditions [107]. 

Ethylene is another growth regulator involved in low K^+^ stress responses [36]. Ethylene triggers upregulation of high-affinity K^+^ transporter HAK5 in low K^+^ environments [36]. Ethylene insensitive mutants, *ein1-1* and *etr1-1* (*ethylene insensitive 1-1*; *ethylene response1-1*), do not show diminished root growth under K^+^ deficiency [36], which is inconsistent with the direct role of K^+^ status on meristematic cells mentioned above. Regulation of HAK5 also relies on ROS [37,77]. K^+^-deprived roots have higher H_2_O_2_ levels [77] and altered expression of key peroxidase RCI3 (RARE COLD INDUCIBLE GENE3), which regulates transcription of *HAK5*. RCI3 is upregulated under low K^+^ stress, but *rci3* knock-out mutants or *RCI3-ox* overexpressing plants have similar root growth when compared to wild-type plants under K^+^ deficiency [37]. On the other hand, reactive nitrogen species NO inhibited lateral root growth in low K^+^ tobacco [90]. In *Arabidopsis thaliana*, NO is involved in iron toxicity-induced disruption of K^+^ homeostasis (reduced K^+^ retention) within the root tip, which results in root growth inhibition [111].

From the presented data we can conclude that a K^+^ shortage modulates auxin transport and RAM maintenance pathways, at least partially through ethylene and NO signaling, and thus decreases the activity of the meristem. However, the complexity of the regulatory network and its hierarchy still await further clarification. Interaction and crosslinking between nutrient response pathways is emerging, but current knowledge is still incomplete. In *Arabidopsis thaliana*, the loss of nitrate transporter NRT1.5/NPF7.3 led to a lower number of lateral roots in low K^+^ plants [112]. NRT1.5 participates in NO_3_^−^ xylem loading [113] and modulates K^+^ homeostasis under low K^+^ via regulation of K^+^ root-to-shoot translocation and coordination of NO_3_^−^/K^+^ absorption [112,114,115]. The exact mechanism linking NRT1.5 with lateral root primordia is unknown. K^+^ shortage in *nrt1.5* roots is one possible explanation [112], but the role of NRT1.5 is not limited to low K^+^ stress only. *NRT1.5/NPF7.3* expression increases under low Pi, and the *nrt1.5* mutant exhibits fewer lateral roots also in low Pi conditions as well [116]. 

## 6. Phloem Transport

Growth of heterotrophic roots relies on the delivery of carbohydrates via the phloem. K^+^ seems to take part in carbohydrate phloem loading and is transported in the phloem in large quantities. After identification of K^+^ retranslocation within the plant body via the phloem [117], K^+^ fluxes were quantified for several plant species and growth conditions. The amount of recirculated K^+^ is usually more than 40% of the root acquired K^+^, carried by the ascending xylem transpiration stream [118,119]; it may rise up to 70% in saline conditions [120,121]. 

Concentration of K^+^ in the phloem serves as a feedback signal for shoot demand of K^+^ [122]. Phloem-supplied K^+^ supports root growth to some extent and its utilization might even exceed K^+^ supply from soil under certain circumstances [118]. Phloem-transported K^+^ could also feed individual roots growing in K^+^ depleted soil patches. However, the physiological relevance of this process is not fully understood. For example, barley plants supplied with radiolabeled K^+^ in split-root culture showed only a minor allocation of the tracer to K-depleted roots compared to shoot [123].

It is generally accepted that K^+^ starvation decreases the rate of photosynthesis and carbohydrate export from source leaves via the phloem, as shown in different plant species [21,22,82,124,125]. Among others, K^+^ enrichment enhanced the rate of phloem exudation in *Ricinus communis* [126,127], increased export of ^14^CO_2_-derived compounds from leaves in sugar beet [128], and increased the velocity of C transfer in the trunk of *Eucalyptus grandis* [129]. Moreover, *Arabidopsis thaliana* plants defective in heavy metal-binding protein NAKR1 (SODIUM POTASSIUM ROOT DEFECTIVE1) over-accumulated K^+^ in the shoots, which results in impaired phloem loading, a smaller root system, and late flowering [130]. These data indicate a crosslink between K^+^ recycling and transport of assimilates, which is important for root growth. *NaKR1* is expressed in phloem companion cells all over the plant and its action is essential for proper K^+^ distribution [130]. NaKR1 also regulates the expression of *FT* (*FLOWERING LOCUS T*) via miR156-SPL3 (SQUAMOSA PROMOTER BINDING PROTEIN-LIKE3) module in a partially K^+^-dependent manner, which indicates a link between K^+^ status, sucrose export, and regulation of flowering. Accordingly, *Arabidopsis thaliana* loss-of-function mutants in AKT2/3, a phloem-specific K^+^ channel, exhibit decreased sucrose phloem loading, reduced K^+^ dependence of phloem membrane potential, and delayed flowering [131,132]. It is proposed that the transmembrane gradient of K^+^ between apoplast and sieve element/companion cell complex acts as an additional energy source (decentralized energy storage) for sucrose transport, which increases in importance under ATP-limited conditions [133]. Post-translational modulation of AKT2/3 channel gating properties modulates K^+^ permeability, which allows the utilization of K^+^ gradient to overcome local energy limitation [133,134]. This described mechanism is in agreement with *AKT2/3* being expressed predominantly in the phloem of photosynthetically-active parts of the shoot; its expression gradually increases after the onset of a light period, and is higher in shaded compared to illuminated leaves [132]. 

## 7. Root System Resistance to Stress

K^+^ supply improves plant resistance to some stress factors. In this chapter we present examples of stresses that limit root growth and are alleviated by K^+^ supply. Among others, sufficient K^+^ availability increases plant salt tolerance. High salinity, mostly caused by NaCl, triggers Na^+^ accumulation and K^+^ loss in plants tissues [135]. Plants tend to keep Na^+^/K^+^ ratio low in autotrophic parts by Na^+^ xylem loading restriction in roots [136], Na^+^ retrieval from xylem sap [137,138], and Na^+^ retranslocation from shoot back to roots [139]. The Na^+^/K^+^ ratio in plants is considered a good measure of the saline stress experienced by the plant [140]. 

Root growth is inhibited by salinity in most plant species [141], but halophytes may exhibit an opposite response [142]. Reduced root elongation or a decreased number of lateral roots are common responses, but mild salinity can increase the number of lateral roots as part of a stress-induced morphogenic response (SIMR) [143]. Preferential allocation of Na^+^ into the root system contributes to salinity-induced root growth inhibition, but seems favorable for long-term survival. *Arabidopsis thaliana hkt1* (*high-affinity K^+^ transporter1*) mutants have lower Na^+^ content in the roots and increased shoot Na^+^ concentration. HKT1 mediates high-affinity K^+^/Na^+^ cotransport and low-affinity Na^+^ uptake [144]. It retrieves Na^+^ from xylem sap and contributes to shoot-to-root Na^+^ retranslocation [139]. Roots of *hkt1* plants grow faster under short-term Na^+^ exposure, but leaves are hypersensitive to salt stress over the course of long-term experiments and root performance decreases over time [137]. 

Management of the Na^+^/K^+^ ratio in roots is crucial to sustaining root growth and enhancing salt tolerance [140]. The *sos2* mutant, one of the *salt overly sensitive* mutants of *Arabidopsis thaliana*, is very sensitive to low K^+^ conditions and exhibits strong root growth inhibition in saline conditions [145]. *SOS2* is not directly involved in K^+^ transport but encodes a serine/threonine protein kinase, which is upregulated under salt stress [146]. SOS2 modulates the activity of H^+^/Na^+^ antiporter SOS1 [147,148] and activates H^+^/Ca^2+^ antiporter CAX1 (CALCIUM EXCHANGER1) [149].

K^+^ supply also alleviates adverse effects of other competing ions (e.g., Cs^+^, NH_4_^+^). Excess NH_4_^+^ leads to cessation of root growth, especially when K^+^ or NO_3_^−^ are scarce [150,151,152]. Activity of the root apical meristem as well as cell volume growth are negatively affected [153,154], comprising a general phenomenon called ammonium toxicity syndrome. This syndrome is caused by multiple mechanisms, such as energy demanding NH_3_ cycling on the plasmatic membrane [150], carbon depletion [155], pH changes [156], and induced starvation of Ca^2+^, Mg^2+^, and K^+^ [157]. K^+^ starvation is an important factor in the syndrome and even a small enhancement in K^+^ accessibility alleviates NH_4_^+^ toxicity [158,159,160]. K^+^ uptake is disrupted under high external NH_4_^+^ levels due to K^+^/NH_4_^+^ competition for K^+^ channels [159,161].

Cs^+^ is another element competing for K^+^ uptake mechanisms. High-affinity K^+^ transporters mediate most of the Cs^+^ uptake, e.g., AtHAK5 in *Arabidopsis thaliana* [105] or OsHAK1 in rice [162]. Cs^+^ is not naturally occurring in soils, but has become present in the environment as a pollutant since the testing of nuclear bombs and the meltdowns of nuclear power plants [163,164]. When concentrated enough, Cs^+^ becomes toxic and inhibits root growth [105]. Similarly to Na^+^, Cs^+^ toxicity depends on the Cs^+^/K^+^ ratio [165], and it is alleviated by K^+^ supply [105,166]. K^+^ outcompetes Cs^+^ in uptake [167] and high-K plants suppress expression of high-affinity transporters [27]. Plant accumulation of Cs^+^ and the risk of its entrance to the food chain is reduced at elevated K^+^ levels [166].

It is generally supposed that an appropriate supply of K^+^ would have a positive influence on biotic stress resistance, but experimental data are highly dependent on host/pest pairs and evaluation methods, with effects ranging from positive to negative (reviewed e.g., [26,82,168]). Enhanced resistance of K^+^ replete plants may reside in the greater energy reserves of plants, increased mechanical resistance of plant bodies, and lower concentrations of simple saccharides and amino acids within tissues [82]. On the other hand, plant defense and K^+^ deficiency overlap in jasmonic acid (JA) and ethylene signaling. Enhancement of plant defenses by K^+^ starvation is therefore possible [82,169]. *Arabidopsis thaliana* accumulates jasmonic acid and glucosinolates under K^+^ starvation [170] and might be more resistant to pests. Enhanced resistance to biotic stress was indeed confirmed for Pi-starved *Arabidopsis thaliana*, which also accumulates jasmonic acid [171]. Methyl jasmonate (bioactive metabolite of JA) strongly inhibits root growth [172], which is in agreement with the general root growth response in most plants under K^+^ or Pi deficiency [79]. These findings suggest that priming by nutrient stress may lead to enhanced resistance to biotic stress in *Arabidopsis thaliana*, although overall root growth might be suppressed.
